# Supplementary material for: Parent-Reported Perceived Cognitive Functioning Identifies Cognitive Problems in Children Who Survived Neonatal Critical Illness
Source: Children (Basel). 2022 Jun 16;9(6):900. doi: 10.3390/children9060900 (PMC9222129; doi:10.3390/children9060900)
Supplement: Supplementary file 1 [file children-09-00900-s001.zip › children-1741257-supplementary.pdf]

children

Supplementary Materials

Supplementary A

Table S1. Overview of neuropsychological assessments.

Domain	Sub Domain	Task	Variable
Intelligence		Wechsler intelligence scales for children, third version for the Netherlands (WISC-III-NL)* [23]	Total Intelligence Quotient (Total IQ)
		Wechsler Adult Intelligence Scale, fourth version for the Netherlands (WAIS-IV-NL) [24, 25]	Total Intelligence Quotient (Total IQ)
Attention	Processing speed	Trail Making Test Section A (TMTA) [27, 28]	TMTA time (TMTA t)
	Selective attention	Stroop color word test [27, 28]	Stroop interference (Stroop IF)
	Sustained attention	Dot Cancellation test (DCT) [30]	Series time (DCT-ST)<br>Standard deviation of the series time (DCT-SD)
Verbal memory	Working Memory	Subtest Digit Span of the WISC/WAIS [23-25]	Total Digit Span
	Immediate and delayed recall	Rey Auditory Verbal Learning Test (RAVLT) [28, 29]	RAVLT total<br>RAVLT recall
Visuospatial memory	Visuospatial Memory	Subtest Spatial Span of the Wechsler Nonverbal Scale of Ability (WNV-SS) [32]	WNV-SS total<br>WNV-SS forward
	Immediate and delayed recall	Rey Complex Figure test (RCFT) [31]	RCFT immediate<br>RCFT delayed<br>RCFT recognition
Executive Functioning	Strategy Formation	Key Search of the Behavioral Assessment of the Dysexecutive Syndrome (BADS-C)[26]	BADS-C Key Search
	Planning (task scheduling and performance monitoring)	Modified Six Elements of the Behavioral Assessment of the Dysexecutive Syndrome (BADS-C) [26]	BADS-C Modified Six Elements
	Cognitive flexibility	Trail Making Test Section B (TMTB) [27, 28]	TMTB time (TMTBt)
	Working Memory	Subtest Spatial Span of the Wechsler Nonverbal Scale of Ability (WNV-SS) [32]	WNV-SS backwards

	Processing Speed	WISC/WAIS Processing Speed Index [23-25]	Processing Speed Index

*: Full form for 8-year-old children. Two-subtest short form (Block Design and Vocabulary) for 12-year-old children.

Table S2. Outcomes of neuropsychological assessments in different subgroups.

		**CDH (*n* = 57)**		**EA (*n* = 54)**	**CLM (*n* = 20)**	**ECMO, non-CDH (*n* = 37)**	***p*-value**
		Non-ECMO (*n* = 46)	ECMO (*n* = 11)				
	Self-reported PedsPCF*	-0.58 (0.71)	0.13 (0.69)	-0.12 (0.88)	-0.19 (1.07)	-0.57 (0.88)	0.036[1]
	Proxy-reported PedsPCF*	-0.42 (1.02)	-0.46 (1.11)	-0.06 (1.02)	-0.55 (1.05)	-0.59 (1.12)	0.125[2]
	Total BRIEF	0.32 (1.00)	0.25 (0.70)	0.58 (1.01)	0.57 (0.88)	-0.14 (0.89)	0.012[1]
	Behavior regulation index	0.26 (0.95)	0.60 (0.79)	0.51 (0.94)	0.63 (0.97)	0.07 (1.02)	0.166[2]
	Metacognition index	0.33 (0.98)	0.03 (0.81)	0.48 (0.99)	0.46 (0.84)	-0.23 (0.84)	0.008[1]
**Intelligence**	Total IQ	104 (17)	93 (24)	103 (15)	107 (15)	90 (17)	0.000[1]
**Attention**	TMTA t	0.45 (0.78)	-0.21 (1.10)	0.40 (1.05)	0.53 (1.07)	0.00 (1.58)	0.306[2]
	Stroop IF	-0.09 (1.22)	-0.64 (1.60)	-0.16 (1.17)	0.04 (1.22)	-0.48 (1.03)	0.341[1]
	DCT-ST	-0.81 (1.28)	-1.75 (1.21)	-0.83 (1.22)	-1.23 (1.20)	-0.86 (1.37)	0.272[2]
	DCT-SD	-1.12 (1.35)	-1.54 (1.54)	-1.14 (1.47)	-0.91 (1.85)	-1.28 (1.62)	0.827[2]
**Verbal Memory**	Digit Span	0.29 (0.82)	-0.03 (0.80)	-0.03 (1.09)	0.31 (0.86)	-0.31 (1.10)	0.057[1]
	RAVLT total	-0.40 (1.21)	-1.13 (1.35)	0.01 (1.13)	0.12 (1.10)	-0.83 (1.32)	0.002[1]
	RAVLT recall	-0.74 (1.38)	-1.48 (1.95)	-0.17 (1.01)	-0.25 (1.22)	-1.16 (1.44)	0.005[2]
**Visuospatial Memory**	WNV-SS total	-0.06 (0.93)	0.07 (0.60)	-0.27 (0.85)	-0.12 (0.89)	-0.44 (1.07)	0.305[1]
	WNV-SS forward	0.07 (1.06)	0.52 (0.61)	-0.16 (0.96)	0.09 (0.98)	-0.35 (1.17)	0.090[1]
	RCFT immediate	-0.68 (1.29)	-0.94 (1.51)	-0.26 (1.02)	-0.60 (1.11)	-0.96 (1.34)	0.088[1]
	RCFT delayed	-0.86 (1.24)	-1.11 (1.56)	-0.37 (1.04)	-0.47 (1.23)	-1.26 (1.33)	0.027[2]
	RCFT recognition	0.08 (1.22)	-0.85 (1.61)	0.09 (1.00)	-0.01 (1.21)	-0.31 (1.26)	0.299[2]
**Executive Functioning**	BADS-C Key Search	0.07 (0.91)	-0.54 (1.25)	0.38 (1.07)	0.52 (1.06)	0.07 (1.23)	0.045[2]
	BADS-C Modified Six Elements	-0.59 (0.67)	-1.13 (1.04)	-0.33 (0.83)	-0.48 (0.69)	-0.42 (0.91)	0.315[1]

TMTB t	0.22 (0.94)	-0.01 (0.97)	0.33 (0.84)	0.43 (0.72)	-0.53 (1.42)	0.006[2]
WNV-SS backwards	-0.13 (0.98)	-0.36 (0.66)	-0.31 (0.78)	-0.22 (0.80)	-0.46 (0.89)	0.836[1]
Processing Speed Index	0.22 (1.02)	-0.16 (1.63)	0.06 (1.01)	0.09 (0.99)	-0.56 (1.21)	0.022[1]

Most data are presented as mean z-scores (*SD*) (mean = 0, *SD* = 1).

Total IQ is presented as standard scores (mean = 100, *SD* = 15).

CDH = congenital diaphragmatic hernia, ECMO = extracorporeal membrane oxygenation, EA = esophageal atresia, CLM = congenital lung malformations, PedsPCF = pediatric perceived cognitive functioning, IQ = Intelligence Quotient, TMTA t = Trail Making Test Section A time, Stroop IF = Stroop interference factor, DCT-ST = Dot Cancellation Test series time, DCT-SD = Dot Cancellation Test standard deviation series time, RAVLT = Rey Auditory Verbal Learning Test, WNV-SS = Weschler Nonverbal Scale of Ability-Spatial Span, RCFT = Rey Complex Figure Test, BADS-C = Behavioral Assessment of the Dysexecutive Syndrome for Children, TMTB t = Trail Making Test Section B time.

*: The 30-item questionnaire.

[1] One-way analysis of variance (ANOVA).

[2] Kruskal–Wallis test.

Table S3. Simple regressions per variable for self-reported PedsPCF.

Variable	Number	B (*SE*)	95% CI of B	R^2	*p*-value
**Total IQ**	133	0.007 (0.005)	-0.002 – 0.016	0.016	0.150
**TMTA t**	133	0.085 (0.072)	-0.057 – 0.227	0.011	0.240
**Stroop IF**	132	0.009 (0.069)	-0.127 – 0.144	0.000	0.899
**DCT-RT**	131	-0.034 (0.061)	-0.156 – 0.087	0.002	0.579
**DCT-SD**	131	-0.010 (0.051)	-0.110 – 0.091	0.000	0.850
**Digit Span**	132	-0.013 (0.081)	-0.173 – 0.148	0.000	0.876
**RAVLT total**	132	0.090 (0.059)	-0.026 – 0.207	0.018	0.128
**RAVLT recall**	132	0.085 (0.054)	-0.021 – 0.191	0.019	0.117
**WNV-SS total**	133	-0.052 (0.087)	-0.225 – 0.121	0.003	0.554
**WNV-SS forwards**	133	0.000 (0.075)	-0.148 – 0.149	0.000	0.997
**RCFT immediate**	133	0.055 (0.062)	-0.068 – 0.178	0.006	0.377
**RCFT delayed**	133	0.057 (0.059)	-0.060 – 0.175	0.007	0.337
**RCFT recognition**	133	-0.028 (0.065)	-0.156 – 0.100	0.001	0.667
**BADS-C Key Search**	131	-0.025 (0.075)	-0.174 – 0.125	0.001	0.746
**BADS-C Modified Six Elements**	84*	-0.148 (0.116)	-0.379 – 0.082	0.020	0.205
**TMTB t**	131	0.055 (0.074)	-0.092 – 0.201	0.004	0.463
**WNV-SS backwards**	133	-0.094 (0.095)	-0.281 – 0.093	0.007	0.323
**Processing Speed Index**	133	0.118 (0.073)	-0.026 – 0.263	0.020	0.108

B (SE) = Unstandardized coefficient β (standard error); 95% CI of B = 95% confidence interval of unstandardized β coefficient.
IQ = Intelligence Quotient, TMTA t = Trail Making Test Section A time, Stroop IF = Stroop interference factor, DCT-ST = Dot Cancellation Test series time, DCT-SD = Dot Cancellation Test standard deviation series time, RAVLT = Rey Auditory Verbal Learning Test, WNV-SS = Wechsler Nonverbal Scale of Ability-Spatial Span, RCFT = Rey Complex Figure Test, BADS-C = Behavioral Assessment of the Dysexecutive Syndrome for Children, TMTB t = Trail Making Test Section B time.
*: Last subtest of the test battery and consequently missing due to lack of time.

Figure S1. Association between 30-item and 10-item PedsPCF. PedsPCF = pediatric perceived cognitive functioning.

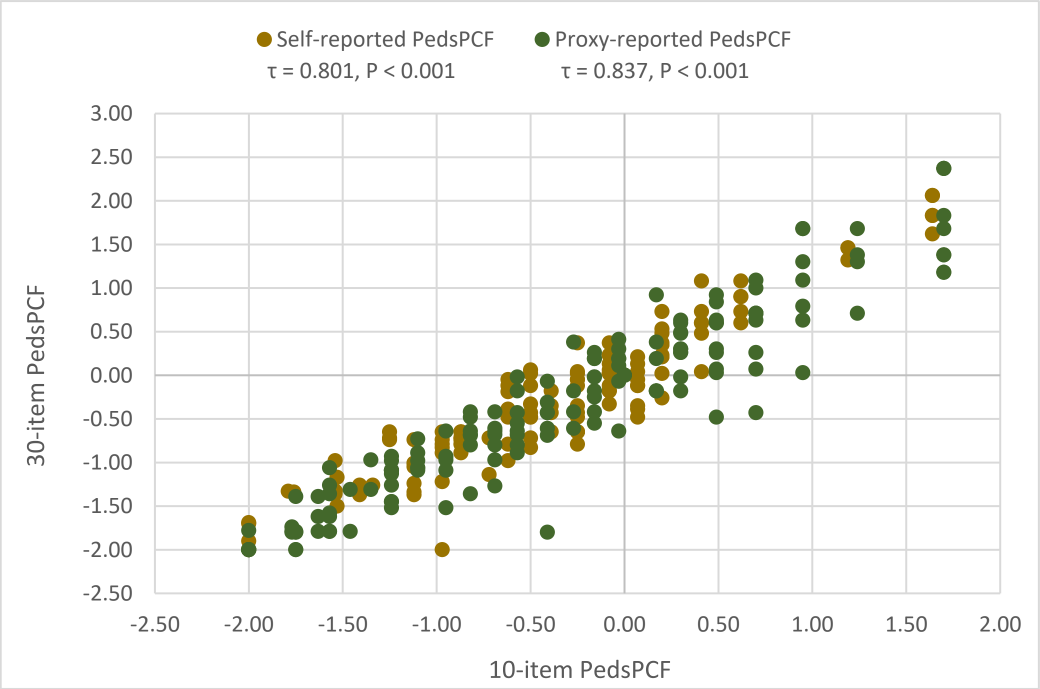

Figure S2. Flowchart triage prior to neuropsychological assessment.

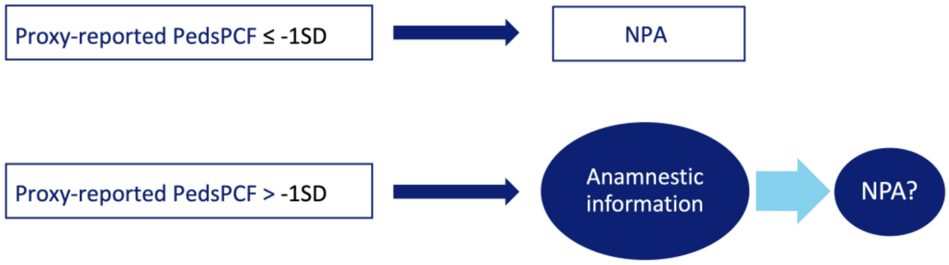

Supplementary B. Descriptions of the neuropsychological assessments.

Content largely reproduced from Schiller et al. [42].

Wechsler Intelligence Scale for Children (WISC-III-NL)

Full form of the WISC-III-NL was used to assess general intelligence at 8 years. A short-form with two subtests, Block Design and Vocabulary, of the WISC-III-NL was used to assess general intelligence at 12 years [43]. The WISC-III-NL has been shown to have good reliability and validity. A normalized population mean of 100 with a standard deviation of 15 was used [23].

Weschler Adult Intelligence Scale (WAIS-IV-NL)

Abbreviated version of the WAIS-IV-NL was used in adolescents of 17 years old to assess general intelligence [24, 25].

Dot Cancellation Test

This paper-and-pencil test measures sustained attention in terms of speed and variability of speed. It consists of a piece of paper on which figures made of three, four, or five dots are displayed in 33 rows. The child is instructed to mark all figures with four dots as precisely and quickly as they can [30].

Stroop Color Word Test (Stroop)

The Stroop consists of three trials: in the first trial (Stroop 1), the subject must read color names; in the second trial (Stroop 2) name printed colors; and in the third trial (Stroop 3) name the colors without being distracted by the word, even if the word represents another color. Selective attention and inhibition are measured with this test, using the difference score between Stroop 3 and Stroop 2 [27,28].

Trail Making Test (TMT)

This paper-and-pencil test consists of two parts. In the first part (part A), the subject must draw lines to connect consecutively numbered circles on a sheet. In the second part (part B), the subject must consecutively but alternately connect numbered and lettered circles on another worksheet. The aim of the test is to finish each part as quickly as possible. This test measures visual conceptual and visuomotor tracking as well as divided attention [27, 28].

WISC-III-NL/WAIS-IV-NL—subtest Digit Span

Digit Span consists of random number sequences that increase in length and that the examiner reads aloud at the rate of 1 number per second. The child has to reproduce these numbers in the same order. Next, the sequences must

be recalled backwards (3-5-7 becomes 7-5-3). The first part of the test measures short-term auditory memory and short-term retention capacity. The second part measures auditory working memory [23-25].

Wechsler Nonverbal Scale of Ability (WNV)—subtest Spatial Span

Spatial Span requires the child to touch a group of blocks arranged on a board in a nonsystematic manner in the same (part 1) and reverse order (part 2) as demonstrated by the examiner. The first part of the test measures short-term visuospatial memory and short-term retention capacity. The second part measures visuospatial working memory [32].

Rey Auditory Verbal Learning Test (RAVLT)

The RAVLT consists of five presentations with recall of a 15-word list, a sixth recall trial after 30 minutes, followed by a recognition trial. This test measures memory span, short- and long-term verbal memory, verbal recognition, and learning curve. Recognition scores are not used in this study [29, 44].

Rey Complex Figure Test (RCFT)

The RCFT consists of three trials. First, the child has to copy a complex figure (copy). Then, after 3 (immediate) and after 30 minutes the figure must be drawn from memory (delayed). Next, different figures are shown, and the child has to indicate whether these figures were in the original figure (recognition). This test measures visual integration, short- and long-term visual-spatial memory, and visual-spatial recognition [27, 45].

Behavioral Assessment of the Dysexecutive Syndrome for Children (BADS-C)

Test battery consisting of several tests measuring executive functioning. Of this battery, two subtests were used:

Key Search

A test of strategy formation. The child is asked to demonstrate how they would search a field for a set of lost keys, and their strategy is scored according to its efficiency and functionality [26].

Modified Six Elements

The child is asked to work on six different tasks for which they have five minutes. The child needs to make sure that by the end of the five minutes, parts of all six tasks have been performed and the child must strive to do as much as possible of each task. This is a test of planning, task scheduling, and performance monitoring [26].

WISC-III-NL/WAIS-IV-NL—Processing Speed Index

This is a composite measure of two subtests of the Wechsler scales for processing speed: Coding and Symbol Search [23-25].

We used Dutch versions of all tests.
